# Molecular Analysis of Prognosis and Immune Infiltration of Ovarian Cancer Based on Homeobox D Genes

**DOI:** 10.1155/2022/3268386

**Published:** 2022-09-29

**Authors:** Buze Chen, Cui Gao, Haihong Wang, Jieyun Sun, Zhengxiang Han

**Affiliations:** ^1^Department of Gynecology, The Affiliated Hospital of Xuzhou Medical University, Xuzhou, 221000 Jiangsu, China; ^2^Xuzhou Medical University, Xuzhou, 221000 Jiangsu, China; ^3^Department of Obstetrics, Jinhu County People's Hospital, Huai'an, 223000 Jiangsu, China; ^4^Department of Oncology, The Affiliated Hospital of Xuzhou Medical University, Xuzhou, 221000 Jiangsu, China

## Abstract

**Background:**

Homeobox D (HOXD) genes were associated with cancer pathogenesis. However, the role of HOXD genes in ovarian cancer (OC) and the possible mechanisms involved are unclear. In this study, we analyzed the function and regulatory mechanisms and functions of HOXD genes in OC based on comprehensive bioinformatics analysis.

**Methods:**

Expression of HOXD1/3/4/8/9/10/11/12/13 mRNA was analyzed between OC tissue and normal tissue using ONCOMINE, GEO, and TCGA databases. The relationship between HOXD expression and clinical stage was studied by GEPIA. The Kaplan-Meier plotter was used to analyze prognosis. cBioPortal was used to analyze the mutation and coexpression of HOXDs. GO and KEGG analyses were performed by the DAVID software to predict the function of HOXD coexpression genes. Immune infiltration analysis was used to evaluate the relationship between the expression of HOXD genes and 24 immune infiltrating cells.

**Results:**

The expression of HOXD3/4/8/9/10/11 was significantly lower in OC tissues than in normal ovarian tissues, while the expression of HOXD1/12/13 was significantly higher in OC tissues. The expression of HOXD genes was associated with FIGO stage, primary therapy outcome, tumor status, anatomic neoplasm subdivision, and age. The expression levels of HOXD1/3/4/8/9/10 correlated with tumor stage. HOXD1/8/9 could be served as ideal biomarkers to distinguish OC from normal tissue. Low HOXD9 expression was associated with shorter overall survival (OS) (HR: 0.75; 95% CI: 0.58–0.98; *P* = 0.034) and progression-free survival (PFS) (HR: 0.69; 95% CI: 0.54–0.87; *P* = 0.002). The HOXD coexpression genes were associated with pathways including cell cycle, TGF-beta signaling pathway, cellular senescence, and Hippo signaling pathway. HOXD genes were significantly associated with immune infiltration.

**Conclusion:**

The expression of HOXD genes is associated with clinical characteristics. HOXD9 is a new biomarker of prognosis in OC, and HOXD1/4/8/9/10 may be potential therapeutic targets. The members of the HOXD genes may be the response to immunotherapy for OC.

## 1. Introduction

Ovarian cancer (OC) is one of the most common gynaecological tumors, ranking fourth in incidence and third in mortality worldwide [1, 2]. In China, OC has the second highest mortality rate among gynaecological tumors and is on the rise, while the incidence is declining [3]. It is difficult to detect at an early stage, and most patients are diagnosed at an advanced stage [4]. Despite advances in the treatment of OC with chemotherapy, radiotherapy, surgery, and targeted therapies, the 5-year OS rate for patients with advanced OC is around 30% [5, 6]. Therefore, there is a need to explore the genetic signature of prognostic prediction associated with the underlying mechanisms of OC progression.

Homeobox genes are regulatory genes that share a common 180-183 bp sequence and encode a 61-amino acid structural domain known as the homeodomain. This homeodomain is a DNA binding domain that functions as a transcription factor [7]. In humans, the HOX genes are divided into four clusters (HOXA, HOXB, HOXC, and HOXD) on different chromosomes [8]. HOXDs contain 9 members, including HOXD1, HOXD3, HOXD4, HOXD8, HOXD9, HOXD10, HOXD11, HOXD12, and HOXD13. HOXD1 inhibited cell proliferation, cell cycle, and TGF-*β* signaling in kidney renal clear cell carcinoma (KIRC) [9]. By identifying the YY1-HOXD3-ITGA2 regulatory axis as a potential therapeutic target for hepatocellular carcinoma (HCC) treatment, a new and complete pathway for HCC treatment is offered [10]. Increased expression of HOXD3 was an independent and important predictor of poor prognosis in breast cancer (BRCA) patients [11]. Overexpression of HOXD4 is significantly associated with poorer prognosis in patients with gastric cancer (GC), suggesting the potential of HOXD4 as a novel clinical predictive biomarker and drug target [12]. HOXD8 may be associated with cisplatin resistance and metastasis in advanced OC [13]. Downregulation of miR-142-5p induced resistance to gefitinib in lung cancer PC9 cells through upregulation of HOXD8 [14]. In summary, some members of HOXDs are closely associated with clinical features and drug resistance of tumors, and their expression levels can be used as predictors of tumor prognosis, metastasis, and response to chemotherapy and targeted therapy. HOXD genes play a role in the pathogenesis of pediatric low-grade gliomas [15]. However, the role of HOXDs in OC is unclear. Studying the prognostic value of HOXDs for patients with OC may help to improve the prediction of clinical prognosis in OC and inform personalized treatment.

In this study, we used a comprehensive bioinformatics analysis to analyze the potential of HOXDs in OC as a predictor of OC prognosis, possible regulatory mechanisms, and relationship with immune infiltration. We hope that our study will be useful for the prognosis of biomarker and treatment of OC.

## 2. Materials and Methods

### 2.1. cBioPortal Analysis

The cBio Cancer Genomics Portal (cBioPortal) (http://cbioportal.org) was applied to study mutations in HOXD genes in OC [16]. Queries for visualization and analysis were performed by entering (1) cancer type: ovarian cancer; (2) 3 selected studies: ovarian serous cystadenocarcinoma (TCGA, Nature 2011), ovarian serous cystadenocarcinoma (TCGA, PanCancer Atlas), and ovarian serous cystadenocarcinoma (TCGA, Firehose Legacy); (3) molecular profile: mutations, structural variants, and copy number alterations; (4) selection of patients/case sets: all samples (1365); and (5) input genes: HOXD1 (ENSG00000128645), HOXD3 (ENSG00000128652), HOXD4 (ENSG00000170166), HOXD8 (ENSG00000175879), HOXD9 (ENSG00000128709), HOXD10 (ENSG00000128710), HOXD11 (ENSG00000128713), HOXD12 (ENSG00000170178), and HOXD13 (ENSG00000128714). After submission of queries, accessions were made including origin studies, mutation profiles, mutation number, overall survival (OS) status, OS (months), disease-free status, and disease-free period (months) tracks.

### 2.2. Differential Expression of HOXDs

ONCOMINE (https://www.oncomine.org/resource/login.html) was used to analyze the levels of HOXD mRNAs in OC tissues and normal tissues [17]. Screening criteria are as follows: *P* < 0.05, fold change > 1.5, and top 10% of gene rank [18].

The analysis was carried out according to the reference [19, 20]. Software: R (version 3.6.3). R package: mainly ggplot2 (for visualization). Molecules: HOXD1/3/4/8/9/10/11/12/13. Data: UCSC XENA (https://xenabrowser.net/datapages/) RNAseq data in TPM (transcripts per million reads) format for TCGA and GTEx processed uniformly by the Toil process [21]. Extracted TCGA (https://www.cancer.gov/about-nci/organization/ccg/research/structural-genomics/tcga) OC and corresponding normal tissue data in GTEx. Data filtering: none. Data transformation: RNAseq data in TPM format and log2 transformed for sample-to-sample expression comparisons. Significance markers: ns, *P* ≥ 0.05; ^∗^, *P* < 0.05; and ^∗∗∗^, *P* < 0.001.

### 2.3. Correlation Heat Map

Correlation between every two genes of HOXDs was assessed using a Pearson's correlation coefficient [16]. Software: R (version 3.6.3). R package: mainly ggplot2 (version 3.3.3). Data: RNAseq data in level 3 HTSeq-FPKM format from the TCGA OC project. Data conversion: RNAseq data in FPKM (fragments per kilobase per million) format were converted to TPM format and log2 transformed. Data filtering: remove control/normal (not all items have control/normal).

### 2.4. The Relationship between HOXDs and Clinical Characteristics of OC

Software: R (version 3.6.3). R package: basic R package. Molecules: HOXD1/3/4/8/9/10/11/12/13. The grouping condition is the median. Data were obtained from the TCGA OC project for RNAseq data in level 3 HTSeq-FPKM format. RNAseq data in FPKM format were converted to TPM format and then log2 transformed.

Expression and correlation analyses of HOXDs were carried out on the GEPIA website (http://gepia.cancer-pku.cn/) [22]. The expression of HOXDs at different clinical stages was generated online.

### 2.5. The Relationship between HOXDs and Prognosis of OC

Using the Kaplan-Meier method, the analysis was carried out according to the reference [18, 23]. Software: R (version 3.6.3). R package: survminer package (for visualization) and survival package (for statistical analysis of survival data). Molecules: HOXD1/3/4/8/9/10/11/12/13. Subgroups: 0-50 vs. 50-100. Prognosis type: OS and progression-free survival (PFS). OS is defined as the time from the beginning to death from any cause. PFS is defined as the time from initiation to the onset of arbitrary tumor progression or the onset of death. Data: RNAseq data and clinical data in level 3 HTSeq-FPKM format from the TCGA OC project. Data filtering: retain data with clinical information. Data conversion: RNAseq data in FPKM format were converted to TPM format and analyzed by grouping them according to molecular expression. Additional data: prognostic data from the reference [24].

The survival curves of HOXD12 were plotted using the online Kaplan-Meier plotter database [25].

### 2.6. Univariate and Multivariate Cox Regression Analysis

Software: R (version 3.6.3). R package: survivor package (version 3.2-10). Statistical methods: Cox regression module. Prognosis type: OS and PFS. Included variables: HOXD1/3/4/8/9/10/11/12/13. Data: RNAseq data in level 3 HTSeq-FPKM format from TCGA OC project. Data conversion: RNAseq data in FPKM format were converted to TPM format and log2 transformed. Supplementary data: prognostic data from the reference [24]. Data filtering: remove control/normal (not all items have control/normal) + keep clinical information.

### 2.7. ROC Curve Analysis

The analysis was carried out according to the reference [18]. Software: R (version 3.6.3). R packages: mainly the pROC package (for analysis) || ggplot2 package. Molecules: HOXD1/3/4/8/9/10/11/12/13. Clinical variables: tumor vs. normal. Data: UCSC XENA RNAseq data in TPM format for TCGA and GTEx processed uniformly by the Toil process [21]. Extracted OC for TCGA and corresponding normal tissue data in GTEx. Data filtering: none. Data transformation: RNAseq data in TPM format and log2 transformed for analysis.

### 2.8. Correlation Analysis for Genes Associated with HOXDs in OC

cBioPortal was also used to analyze the relationship between the mutation of HOXDs and survival in OC. Coexpression levels were calculated according to the online instructions of “Similar Genes” part of GEPIA2 (http://gepia2.cancer-pku.cn/index.html#example#e3). The first 100 coexpressed genes were kept separately for each gene, and finally, all coexpressed genes were summarized. To further validate the accuracy of the ONCOMINE and TCGA databases, OC samples from the GEO database were downloaded for analysis [26]. 10 ovarian cancer tumor tissues and 10 normal ovarian tissues contained in GSE29450 were used for differential gene expression analysis.

### 2.9. GO and KEGG Analyses

DAVID database was used to do Gene Ontology (GO) and Kyoto Encyclopedia of Genes and Genomes (KEGG) analyses for the coexpression genes of HOXDs, including BP (biological process), MF (molecular function), CC (cellular component), and pathway analysis [23, 27].

### 2.10. Correlation between the Expression of HOXD Genes in OC and Immune Cells

The analysis was carried out according to the reference [20]. Software: R (version 3.6.3). R package: GSVA package (version 1.34.0) [28]. Immunoinfiltration algorithm: ssGSEA (built-in algorithm of the GSVA package). Molecules: HOXD1/3/4/8/9/10/11/12/13. Immune cells: aDC (activated DC); B cells; CD8 T cells; cytotoxic cells; DC; eosinophils; iDC (immature DC); macrophages; mast cells; neutrophils; NK CD56bright cells; NK CD56dim cells; NK cells; pDC (plasmacytoid DC); T cells; T helper cells; Tcm (T central memory); Tem (T effector memory); Tfh (T follicular helper); Tgd (T gamma delta); Th1 cells; Th17 cells; Th2 cells; and Treg. Data: RNAseq data in level 3 HTSeq-FPKM format from TCGA OC project. Data conversion: RNAseq data in FPKM format were converted to TPM format and log2 transformed. Data filtering: control/normal removed (not all items have control/normal). Other data: markers for 24 immune cells were obtained from the reference [29].

### 2.11. Statistical Analysis

The methodology of our analysis follows the previous literature [18]. The expression of HOXDs between OC tissue and normal ovarian tissue was analyzed using the Wilcoxon rank sum test. *P* < 0.05 were considered statistically significant.

## 3. Results

### 3.1. HOXD Gene Alterations and mRNA Expression in OC

**Figure 1:**
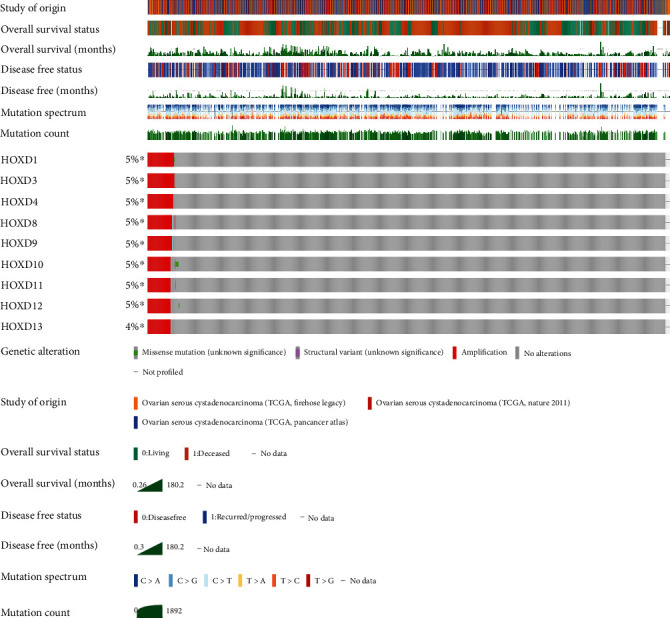
The genetic alteration of HOXD genes in OC by cBioPortal.

**Figure 2:**
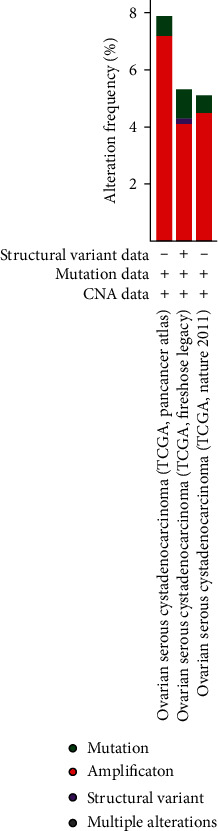
Percentage of HOXD genes in OC cases calculated using the cancer type summary in cBioPortal.

The cBioPortal online tool was used to analyze the gene expression of HOXD genes in OC patients. Alterations in the HOXD genes in OC ranged from 4% to 5% (Figure 1). The structural variation data, mutation data, and CNA (copy number alteration) data from the 3 studies are depicted in Figure 2.

**Figure 3:**
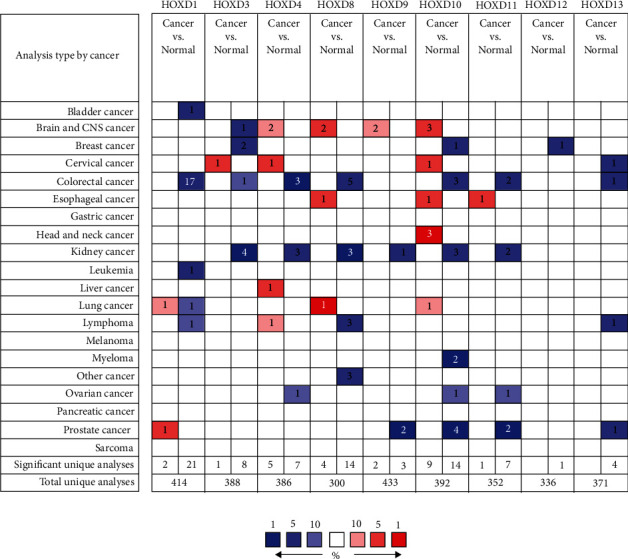
Changes in HOXD mRNA expression between different types of cancer and normal tissues using the ONCOMINE database. Cell color is determined by the best gene rank percentile for the analyses within the cell. Red indicates an increase in expression, blue indicates a decrease in expression, and white indicates that the copy number is neutral. The data in the middle of the square represents the number of data sets.

**Figure 4:**
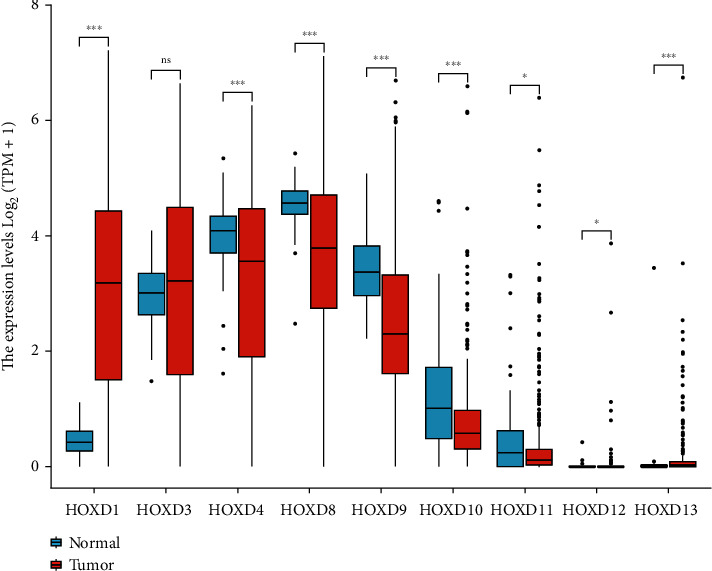
The expression of HOXDs in normal ovarian tissue was compared with that of the OC tissues. Significance markers: ns, *P* ≥ 0.05; ^∗^, *P* < 0.05; and ^∗∗∗^, *P* < 0.001.

As shown in Figure 3 and Table 1, the expression of HOXD4/10/11 mRNA in OC tissues was significantly lower than that in normal ovarian tissues (*P* < 0.05). Among them, HOXD10 had the highest expression change (fold change = 21.976, *P* < 0.05), and 2 data sets confirmed this [30, 31]. As shown in Figure 4, the HOXD1 expression in OC tissues was significantly higher than that in normal ovarian tissues (3.024 ± 0.087 vs. 0.458 ± 0.028, *P* < 0.001), the HOXD4 expression in OC tissues was significantly lower than that in normal ovarian tissues (3.185 ± 0.081 vs. 3.976 ± 0.064, *P* < 0.001), the HOXD8 expression in OC tissues was significantly lower than that in normal ovarian tissues (3.670 ± 0.066 vs. 4.538 ± 0.044, *P* < 0.001), the HOXD9 expression in OC tissues was significantly lower than that in normal ovarian tissues (2.582 ± 0.061 vs. 3.438 ± 0.065, *P* < 0.001), the HOXD10 expression in OC tissues was significantly lower than that in normal ovarian tissues (0.790 ± 0.039 vs. 1.238 ± 0.103, *P* < 0.001), the HOXD11 expression in OC tissues was significantly lower than that in normal ovarian tissues (0.376 ± 0.039 vs. 0.460 ± 0.073, *P* = 0.026), the HOXD12 expression in OC tissues was significantly higher than that in normal ovarian tissues (0.032 ± 0.012 vs. 0.007 ± 0.005, *P* = 0.041), the HOXD13 expression in OC tissues was significantly higher than that in normal ovarian tissues (0.151 ± 0.024 vs. 0.053 ± 0.039, *P* < 0.001), and there was no significant difference in HOXD3 between OC tissues and normal ovarian tissues (3.058 ± 0.082 vs. 2.980 ± 0.052, *P* = 0.262). The mRNA expression levels of HOXD1/12/13 in OC tissues were significantly higher than that in normal ovarian tissues, and the mRNA expression levels of HOXD4/8/9/10/11 in OC tissues were significantly lower than those in normal ovarian tissues. There was no significant difference in HOXD3. As shown in Table 2, compared with normal ovarian tissues, HOXD3 was significantly lower expressed in OC tumor tissues (fold change = 0.331, *P* = 0.026), HOXD4 was significantly lower expressed in OC tumor tissues (fold change = 0.155, *P* < 0.001), HOXD8 was significantly lower expressed in OC tumor tissues (fold change = 0.280, *P* < 0.001), HOXD9 was significantly lower expressed in OC tumor tissues (fold change = 0.373, *P* = 0.009), and HOXD12 was significantly higher expressed in OC tumor tissues (fold change = 6.720, *P* < 0.001). There was no significant difference in HOXD1/10/11/13. The above results from different databases showed that the expression of HOXD4/8/9/10/11 was significantly lower in OC tissues than in normal ovarian tissues, while the expression of HOXD1/12/13 was significantly higher in OC tissues than in normal ovarian tissues.

**Figure 5:**
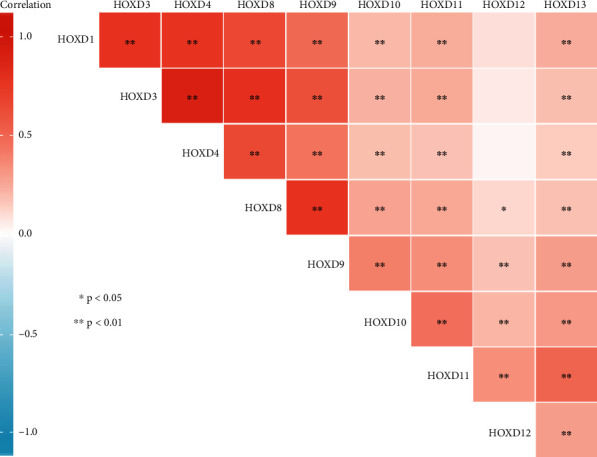
Correlation between every two genes of HOXD genes.

We examined the correlation between HOXD genes using the Pearson correlation analysis. As shown in Figure 5, there was no significant correlation between HOXD12 and HOXD1/3/4. Other HOXD genes were significantly positively correlated with each other.

### 3.2. Relationship between HOXD mRNA Expression and the Clinical Stage and Prognosis of OC

**Figure 6:**
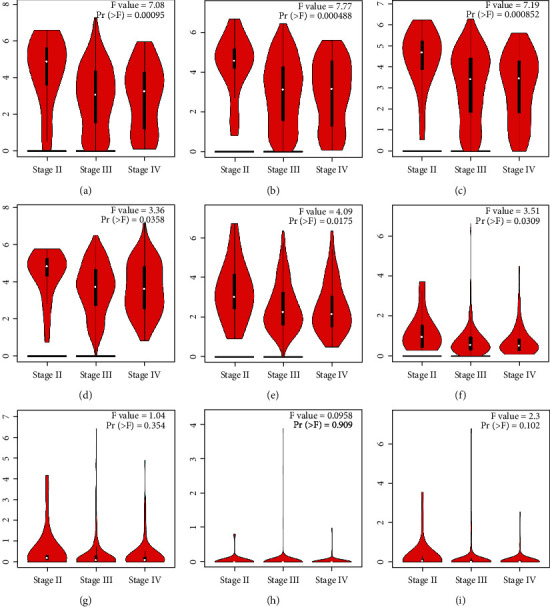
Expression of HOXDs in OC patients at different tumor stages (GEPIA). (a) HOXD1, (b) HOXD3, (c) HOXD4, (d) HOXD8, (e) HOXD9, (f) HOXD10, (g) HOXD11, (h) HOXD12, and (i) HOXD13 were analyzed in this study.

As shown in Figure 6, HOXD1/3/4/8/9/10 were negatively correlated with the clinical stage of OC. HOXD1/3/4/8/9/10 may be closely related to the development of OC. As shown in Table S1, in the TCGA database, the clinical information of 379 OC patients was used for prognostic analysis of HOXD genes. The clinical characteristics included FIGO stage, primary therapy outcome, race, age, histologic grade, anatomic neoplasm subdivision, venous invasion, lymphatic invasion, tumor residual, tumor status, and age. As shown in Table S2, high expression of HOXD1 was associated with FIGO stage (*P* = 0.004), low expression of HOXD3 was associated with FIGO stage (*P* = 0.002) and histological grade (*P* = 0.039), low expression of HOXD4 was associated with FIGO stage (*P* = 0.005), low expression of HOXD8 was associated with FIGO stage (*P* = 0.002), low expression of HOXD9 was associated with FIGO stage (*P* = 0.017), primary therapy outcome (*P* = 0.048), and tumor status (*P* = 0.003), low expression of HOXD10 was associated with anatomic neoplasm subdivision (*P* = 0.028), low expression of HOXD11 was associated with age (*P* = 0.032), and high expression of HOXD13 was associated with FIGO stage (*P* = 0.005).

**Figure 7:**
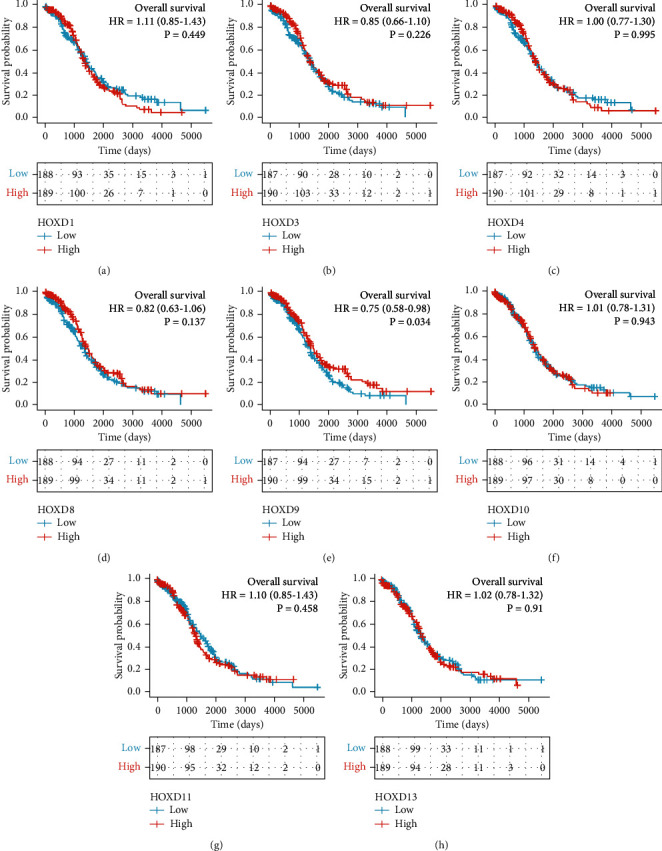
The expression of HOXDs is associated with poor OS in patients with OC. (a) HOXD1, (b) HOXD3, (c) HOXD4, (d) HOXD8, (e) HOXD9, (f) HOXD10, (g) HOXD11, and (h) HOXD13 were analyzed in this study.

**Figure 8:**
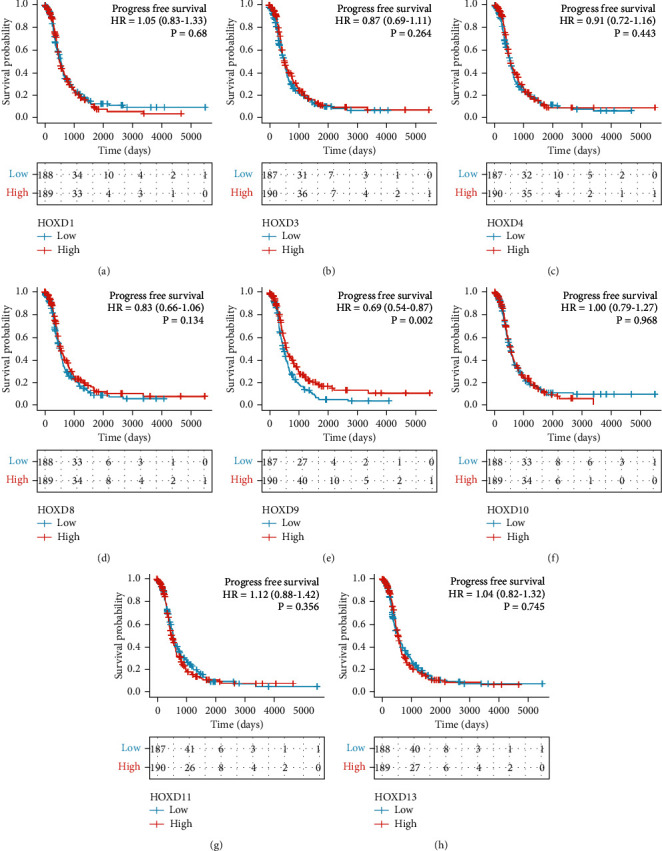
The expression of HOXDs is associated with poor PFS in patients with OC. (a) HOXD1, (b) HOXD3, (c) HOXD4, (d) HOXD8, (e) HOXD9, (f) HOXD10, (g) HOXD11, and (h) HOXD13 were analyzed in this study.

As shown in Figure 7 and Figure S1A, low HOXD9 expression was associated with shorter overall survival (OS) (HR: 0.75; 95% CI: 0.58–0.98; *P* = 0.034) and progression-free survival (PFS) (HR: 0.69; 95% CI: 0.54–0.87; *P* = 0.002). Low HOXD12 expression was associated with shorter OS (HR: 0.86; 95% CI: 0.75–1; *P* = 0.049) and PFS (HR: 0.78; 95% CI: 0.68–0.89; *P* = 0.00023). The OS of the HOXD9/12 mRNA lower expression group was lower than that of the HOXD9/12 low expression group at all time points. It suggested that HOXD9 were risk factors of OC. As shown in Figure 8 and Figure S1B, HOXD9 and HOXD12 low expressions were associated with PFS shortening. The mRNA of HOXD9 can be used as indicators for predicting OC/PFS progression. As shown in Table 3, HOXD9 (HR: 0.754; 95% CI: 0.581-0.978; *P* = 0.034) was independently correlated with OS in multivariate analysis. As shown in Table 4, HOXD9 (HR: 0.690; 95% CI: 0.544-0.875; *P* = 0.002) was independently correlated with OS in multivariate analysis.

### 3.3. Diagnostic Value of HOXD Gene Expression in OC

**Figure 9:**
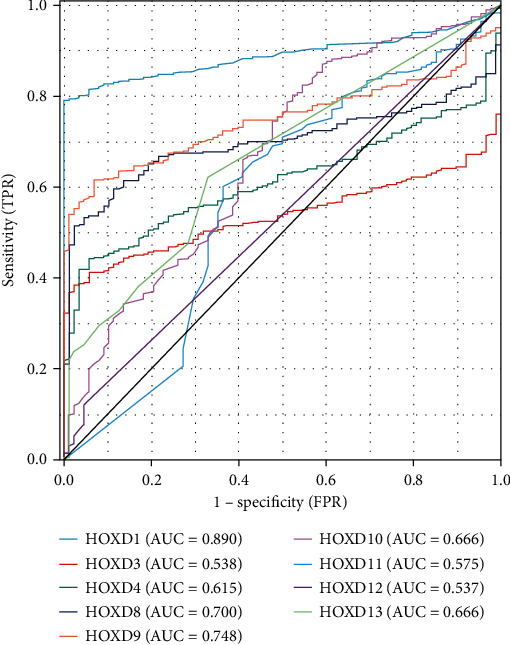
ROC curve showed the efficiency of HOXD expression level in distinguishing OC tissue from nontumor tissues.

As shown in Figure 9, the area under curve (AUC) of HOXD1 was 0.890, the AUC of HOXD3 was 0.538, the AUC of HOXD4 was 0.615, the AUC of HOXD8 was 0.700, the AUC of HOXD9 was 0.748, the AUC of HOXD10 was 0.666, the AUC of HOXD11 was 0.575, the AUC of HOXD12 was 0.537, and AUC of HOXD3 was 0.666. The above results suggest that the expression of HOXD1/8/9 showed good classification efficiency (AUC > 0.7) in OC patients and healthy individuals, indicating that HOXD1/8/9 can be used as biomarkers for OC.

### 3.4. The Function of Genes Associated with HOXD Genes

**Figure 10:**
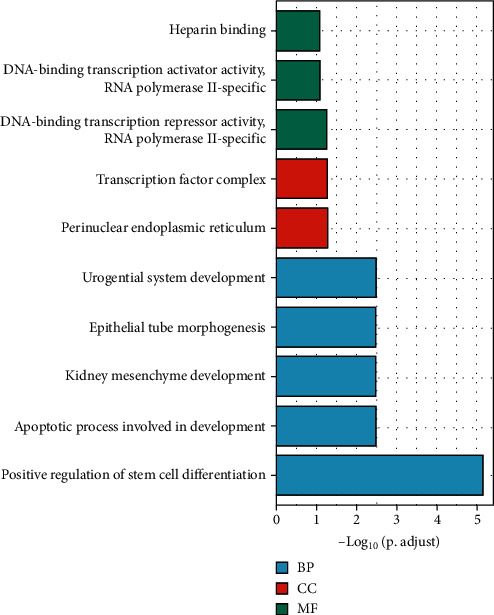
GO analysis of HOXD coexpression genes predicted by DAVID. BP: biological process; MF: molecular function; CC: cellular component.

**Figure 11:**
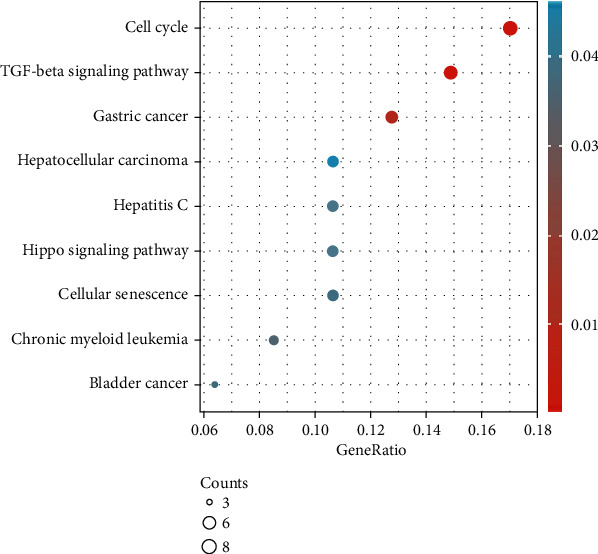
KEGG analysis of HOXD coexpression genes predicted by DAVID.

Some proteins were closely related to the HOXDs (Table S3). These results suggested that changes in the expression profile of HOXDs contributed to the development of OC. The results contained 139 biological processes, mainly including positive regulation of stem cell differentiation, apoptotic process involved in development, kidney mesenchyme development, epithelial tube morphogenesis, and urogenital system development (Figure 10 and Table S4). The 3 enriched molecular functions included DNA-binding transcription repressor activity, RNA polymerase II-specific, DNA-binding transcription activator activity, RNA polymerase II-specific, and heparin binding (Figure 10 and Table S4). The results contained 2 cell components, which were mainly related to perinuclear endoplasmic reticulum and transcription factor complex (Figure 10 and Table S4). The analysis of these functions provides further insight into the cellular localization, geometric distribution, and functional classes of the HOXDs. KEGG analysis showed that 9 pathways, including cell cycle, TGF-beta signaling pathway, gastric cancer, chronic myeloid leukemia, bladder cancer, cellular senescence, Hippo signaling pathway, hepatitis C, and hepatocellular carcinoma, in OC were associated with HOXDs (Figure 11 and Table S4). These results contributed to the study of the mechanism of action of HOXDs in the development of OC and the possibilities for clinically targeted therapy.

### 3.5. Correlation of HOXD Gene Expression and Immune Cells in OC

**Figure 12:**
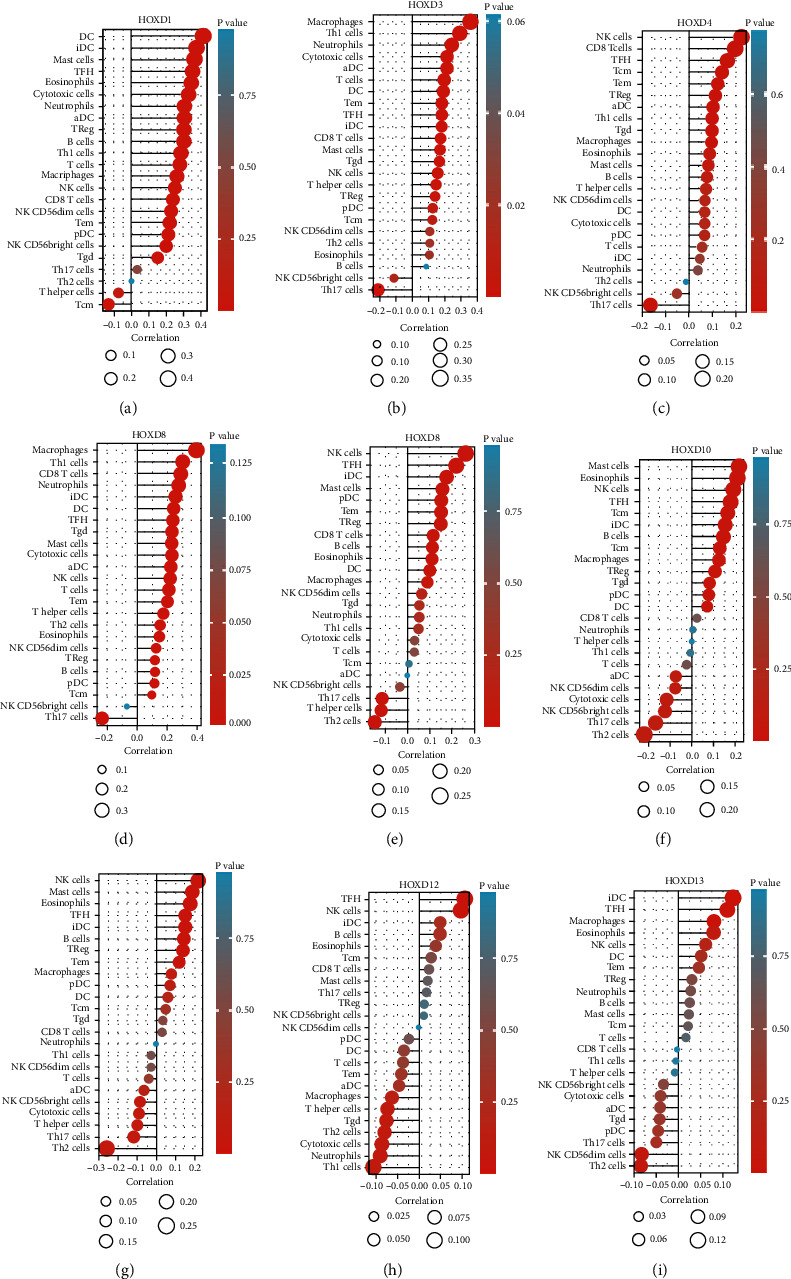
Correlation between the expression of each HOXD gene and the 24 TIICs of OC (lollipop plot). (a) HOXD1, (b) HOXD3, (c) HOXD4, (d) HOXD8, (e) HOXD9, (f) HOXD10, (g) HOXD11, (h) HOXD12, and (i) HOXD13. In the color bar, the darker the color, the smaller the *P* value, indicating a higher statistical significance. The bubble size represents the correlation value; the larger the bubble, the larger the correlation value.

As shown in Figure 12, there was a correlation between HOXD gene expression and immune cells in OC. HOXD1 gene expression was positively correlated with some TIICs, including aDC, cytotoxic cells, DC, iDC, macrophages, neutrophils, NK CD56bright cells, NK CD56dim cells, T cells, T helper cells, Tcm, Tem, TFH, Th1 cells, Th17 cells, and TReg, and negatively correlated with NK cells. HOXD3 gene expression was positively correlated with some TIICs, including aDC, DC, NK CD56bright cells, NK CD56dim cells, T helper cells, Tcm, Tem, and Th1 cells. HOXD4 gene expression was positively correlated with some TIICs, including aDC, cytotoxic cells, DC, NK CD56bright cells, NK CD56dim cells, pDC, T helper cells, Tcm, Tem, Th1 cells, and TReg. HOXD8 gene expression was positively correlated with some TIICs, including aDC, DC, NK CD56dim cells, and Tem. HOXD9 gene expression was positively correlated with some TIICs, including aDC, DC, NK CD56dim cells, Tem, TFH, Th2 cells, and TReg. HOXD10 gene expression was positively correlated with some TIICs, including DC, iDC, macrophages, neutrophils, T helper cells, Tem, and TFH. HOXD11 gene expression was positively correlated with some TIICs, including B cells, CD8 T cells, DC, eosinophils, iDC, macrophages, mast cells, neutrophils, NK CD56dim cells, T cells, T helper cells, Tem, TFH, Tgd, Th1 cells, Th2 cells, and TReg. HOXD12 gene expression was positively correlated with some TIICs, including macrophages, T helper cells, Th2 cells, and TReg. HOXD13 gene expression was positively correlated with some TIICs, including iDC, macrophages, neutrophils, NK CD56dim cells, NK cells, T helper cells, Tcm, Tem, TFH, Tgd, Th1 cells, Th2 cells, and TReg, and negatively correlated with Th17 cells.

## 4. Discussion

HOXDs play an important role in the development, metastasis, and prognosis of various tumors, but the mechanisms are complex. This study used bioinformatics tools to investigate the relationship between HOXDs and the development and prognosis of OC. The results suggested that members of HOXDs could be used as new therapeutic targets and predictive markers for OC. HOXD dysregulation has been reported in many cancers.

HOXD4 protein expression may be associated with poorer prognosis in ovarian serous carcinoma [32]. miR-5692a has oncogenic effects in HCC by targeting HOXD8, which may shed new light on new therapeutic targets and biomarkers for HCC [33]. HOXD1 was lowly expressed in KIRC and correlates with patient OS, DFS, and advanced tumor stage [9]. HOXD9 is upregulated in cervical cancer species, is strongly associated with metastasis rate and poor prognosis in cervical cancer patients, and stimulates the migration and invasive ability of cervical cancer cells by positively regulating HMCN1 levels [34]. Reduced HOXD10 expression promotes a proliferative and aggressive phenotype of prostate cancer [35]. The miR-224/HOXD10 axis may be useful as a promising biomarker and therapeutic approach for the control of NSCLC cell metastasis [36]. HOXD11 may be used as a candidate biomarker for the clinical application of targeted drugs and prognostic assessment therapy for glioma [37]. Progesterone receptor positive cancer tissues have higher levels of HOXD12 and D13 than negative cancer tissues in BRCA [38]. Downregulation of HOXD13 may be a potentially useful prognostic marker for BCRA patients [39]. In this study, the mRNA expression levels of HOXD1/12/13 in OC tissues were significantly higher than that in normal ovarian tissues, and the mRNA expression levels of HOXD3/4/8/9/10/11 in OC tissues were significantly lower than that in normal ovarian tissues. The expression of HOXD genes was associated with FIGO stage, primary therapy outcome, tumor status, anatomic neoplasm subdivision, and age. HOXD1/3/4/8/9/10 was negatively correlated with the clinical stage of OC. ROC analysis results suggested that HOXD1/8/9 could be served as ideal biomarkers to distinguish OC from normal tissue. The HOXD9 low expression was associated with OS/PFS shortening.

The lncRNA insulin-like growth factor 2 antisense RNA (IGF2-AS) is predicted to exert a tumor suppressive effect by HOXD1 [40]. HOXD3 plays a key role in BRCA stemness and drug resistance through integrin *β*3-mediated Wnt/*β*-catenin signaling [41]. HOXD3 promotes the growth of colorectal cancer (CRC) cells and plays a key role in the development and survival of malignant human CRC cells [42]. miRNA-10a inhibits the expression of HOXD4 in human BRCA cells [43]. HOXD8 upregulates caspases 6 and 7 and cleaves PARP, thereby inducing apoptotic events in CRC cells [44]. HOXD9-RUFY3 axis was associated with the development and progression of GC [45]. HOXD9 promotes a malignant biological process in GC, which could be a potential therapeutic target for GC [46]. HOXD10 was inhibited in colon adenocarcinoma cells, thereby downregulating the RHOC/AKT/MAPK pathway to enhance apoptosis and restrain proliferation, migration, and invasion of colon cancer cells [47]. Downregulation of HOXD10 expression by miR-10b overexpression may induce an increase in prometastatic gene products, such as MMP14 and RHOC, and contribute to the acquisition of a metastatic phenotype by epithelial ovarian cancer cells [48]. POU2F1 activity regulates HOXD10 and HOXD11 to promote proliferative and invasive phenotypes in head and neck cancer [49]. GALNT10 can regulate the proliferation and migratory capacity of GC cells by enhancing the expression of HOXD13 and decreasing the sensitivity to 5-Fu [50]. miR-7156-3p regulates stemness, invasion, and growth of glioma cells by mediating HOXD13 [51]. In this study, KEGG analysis showed that HOXDs were related to pathways including cell cycle, TGF-beta signaling pathway, chronic myeloid leukemia, Hippo signaling pathway, HTLV-I infection, microRNAs in cancer, and signaling pathways regulating pluripotency of stem cells in OC.

Immune infiltration and antitumor immune evasion are key mechanisms of tumor progression [19]. HOXD13 was negatively associated with Th17 cells. HOXD1/3/4/8/9/10/11/12/13 were positively associated with other T cells. The emergence of adaptive T cell-based oncology therapies, such as chimeric antigen receptor T cell therapy, may be a promising paradigm for OC, and a better understanding of HOXDs could improve treatment strategies.

This study integrates information on expression levels, mutations, and immune responses to identify potential biomarkers and alterations of HOXD genes in OC. The results promote the understanding of the complex impact of HOXD genes on OC and may help improve clinical decision making. The present study has some limitations in that no in vitro or in vivo experiments were performed to validate the identified role of HOXD genes in OC, which should be attempted in future studies.

## 5. Conclusion

The expression of HOXD genes is associated with clinical characteristics. Downregulation of HOXD9 is an independent factor in the poorer prognosis of OC. HOXDs were key players in mediating OC development and progression through multiple pathways, including regulating immune cells and cell cycle, TGF-beta signaling pathway, cellular senescence, and Hippo signaling pathway. The findings suggested that HOXD9 was a new marker of OC prognosis, while HOXD1/4/8/9/10 may be potential targets for the treatment of OC. The members of the HOXD genes may be the response to immunotherapy for OC.

## Figures and Tables

**Table 1:** Differential expression of HOXD mRNA in OC and ovarian tissues (ONCOMINE database).

HOXD	Types of ovarian cancer vs. ovarian	*P* value	*t*-test	Fold change
HOXD1	NA	NA	NA	NA
HOXD3	NA	NA	NA	NA
HOXD4	Ovarian carcinoma vs. normal	5.30E-7	-8.042	-2.368
HOXD8	NA	NA	NA	NA
HOXD9	NA	NA	NA	NA
HOXD10	Ovarian serous adenocarcinoma vs. normal	8.14E-9	-8.709	-21.976
HOXD11	Ovarian serous adenocarcinoma vs. normal	1.63E-7	-6.975	-15.753
HOXD12	NA	NA	NA	NA
HOXD13	NA	NA	NA	NA

**Table 2:** Differential expression of HOXD mRNA in OC and ovarian tissues (GEO29450).

Gene name	Fold change	*P* value
HOXD1	1.128	0.745
HOXD3	0.331	0.026
HOXD4	0.155	<0.001
HOXD8	0.280	<0.001
HOXD9	0.373	0.009
HOXD10	0.485	0.115
HOXD11	0.777	0.643
HOXD12	6.720	<0.001
HOXD13	1.935	0.171

**Table 3:** Univariate and multivariate Cox regression analyses of HOXDs associated with OS.

Characteristics	Total (*N*)	Univariate analysis		Multivariate analysis	
		HR (95% CI)	*P* value	HR (95% CI)	*P* value
HOXD1 (low vs. high)	377	1.105 (0.853-1.433)	0.449		
HOXD3 (low vs. high)	377	0.853 (0.658-1.104)	0.226		
HOXD4 (low vs. high)	377	1.001 (0.772-1.297)	0.995		
HOXD8 (low vs. high)	377	0.822 (0.634-1.064)	0.137		
HOXD9 (low vs. high)	377	0.754 (0.581-0.978)	0.034	0.754 (0.581-0.978)	0.034
HOXD10 (low vs. high)	377	1.009 (0.779-1.308)	0.943		
HOXD11 (low vs. high)	377	1.103 (0.852-1.429)	0.458		
HOXD13 (low vs. high)	377	1.015 (0.784-1.315)	0.91		

**Table 4:** Univariate and multivariate Cox regression analyses of HOXDs associated with PFS.

Characteristics	Total (*N*)	Univariate analysis		Multivariate analysis	
		HR (95% CI)	*P* value	HR (95% CI)	*P* value
HOXD1	377	1.051 (0.830-1.332)	0.680		
HOXD3	377	0.874 (0.690-1.107)	0.264		
HOXD4	377	0.911 (0.719-1.155)	0.443		
HOXD8	377	0.835 (0.659-1.057)	0.134		
HOXD9	377	0.690 (0.544-0.875)	0.002	0.690 (0.544-0.875)	0.002
HOXD10	377	1.005 (0.793-1.273)	0.968		
HOXD11	377	1.118 (0.882-1.416)	0.356		
HOXD13	377	1.040 (0.821-1.318)	0.745		

## Data Availability

The data analyzed during the current study are available in the TCGA database with the accession number TCGA-OC (Ovarian Serous Cystadenocarcinoma). The data analyzed during the current study are available in the GEO database with the accession number GSE29450. The data used to support the findings of this study are included within the article.

## Supplementary Materials

Supplementary 1 Figure S1: the prognostic value of mRNA level of HOXD12 in OC patients (Kaplan-Meier plotter). (A) Overall survival of HOXD12 and (B) progression free interval of HOXD12. The HR and log-rank *P* values are indicated at the right corner of the plot. Log − rank < 0.05 indicates that the difference is statistically significant.

Supplementary 2 Table S1: patient characteristics in TCGA for OC.

Supplementary 3 Table S2: correlation of HOXD genes with clinical characteristics of OC patients.

Supplementary 4 Table S3: the list of HOXD coexpression genes.

Supplementary 5 Table S4: GO and KEGG analyses of HOXD coexpression genes.

## References

[1] Bray F., Ferlay J., Soerjomataram I., Siegel R. L., Torre L. A., Jemal A. (2018). Global cancer statistics 2018: GLOBOCAN estimates of incidence and mortality worldwide for 36 cancers in 185 countries. *CA: a Cancer Journal for Clinicians*.

[2] Hidayat Y. M., Munizar H. A. B., Winarno G. N. A., Hasanuddin S. S. (2020). Chemokine ligand 5 to predict optimal cytoreduction in ovarian cancer. *International Journal of General Medicine*.

[3] Chen W., Zheng R., Baade P. D. (2016). Cancer statistics in China, 2015. *CA: A Cancer Journal for Clinicians*.

[4] Cannistra S. A. (2004). Cancer of the ovary. *New England Journal of Medicine*.

[5] Kreienbring K., Franz A., Richter R. (2018). The role of PAR1 autoantibodies in patients with primary epithelial ovarian cancer. *Anticancer Research*.

[6] du Bois A., Reuss A., Pujade-Lauraine E., Harter P., Ray-Coquard I., Pfisterer J. (2009). Role of surgical outcome as prognostic factor in advanced epithelial ovarian cancer: a combined exploratory analysis of 3 prospectively randomized phase 3 multicenter trials: by the Arbeitsgemeinschaft Gynaekologische Onkologie Studiengruppe Ovarialkarzinom (AGO-OVAR) and the Groupe d'Investigateurs Nationaux Pour les Etudes des Cancers de l'Ovaire (GINECO). *Cancer*.

[7] McGinnis W., Krumlauf R. (1992). Homeobox genes and axial patterning. *Cell*.

[8] Boncinelli E., Somma R., Acampora D. (1988). Organization of human homeobox genes. *Human Reproduction*.

[9] Cui Y., Zhang C., Li Y., Ma S., Cao W., Guan F. (2021). HOXD1 functions as a novel tumor suppressor in kidney renal clear cell carcinoma. *Cell Biology International*.

[10] Wang L., Gao Y., Zhao X. (2020). HOXD3 was negatively regulated by YY1 recruiting HDAC1 to suppress progression of hepatocellular carcinoma cells via ITGA2 pathway. *Cell Proliferation*.

[11] Shaoqiang C., Yue Z., Yang L. (2013). Expression of HOXD3 correlates with shorter survival in patients with invasive breast cancer. *Clinical & Experimental Metastasis*.

[12] Liu H., Tian H., Zhao J., Jia Y. (2019). High HOXD4 protein expression in gastric adenocarcinoma tissues indicates unfavorable clinical outcomes. *Saudi Journal of Gastroenterology*.

[13] Sun P., Song Y., Liu D. (2018). Potential role of the HOXD8 transcription factor in cisplatin resistance and tumour metastasis in advanced epithelial ovarian cancer. *Scientific Reports*.

[14] Zhu W., Wang J. P., Meng Q. Z., Zhu F., Hao X. F. (2020). MiR-142-5p reverses the resistance to gefitinib through targeting HOXD8 in lung cancer cells. *European Review for Medical and Pharmacological Sciences*.

[15] Buccoliero A. M., Castiglione F., Rossi Degl'Innocenti D. (2009). Hox-D genes expression in pediatric low-grade gliomas: real-time-PCR study. *Cellular and Molecular Neurobiology*.

[16] Chen Y., Li D., Wang D., Peng H. (2022). Comprehensive analysis of Distal-Less homeobox family gene expression in colon cancer.

[17] Sun C. C., Li S. J., Hu W. (2019). RETRACTED: Comprehensive analysis of the expression and prognosis for E2Fs in human breast cancer. *Molecular Therapy*.

[18] Chen J., Tang H., Li T. (2021). Comprehensive analysis of the expression, prognosis, and biological significance of OVOLs in breast cancer. *International Journal of General Medicine*.

[19] Xue D., Li D., Dou C., Li J. (2021). A comprehensive bioinformatic analysis of NOTCH pathway involvement in stomach adenocarcinoma. *Disease Markers*.

[20] Xu K., Wu C.-l., Wang Z.-x. (2021). VEGF family gene expression as prognostic biomarkers for Alzheimer’s disease and primary liver cancer. *Computational and Mathematical Methods in Medicine*.

[21] Vivian J., Rao A. A., Nothaft F. A. (2017). Toil enables reproducible, open source, big biomedical data analyses. *Nature biotechnology*.

[22] Tang Z., Li C., Kang B., Gao G., Li C., Zhang Z. (2017). GEPIA: a web server for cancer and normal gene expression profiling and interactive analyses. *Nucleic Acids Research*.

[23] Zhang J., Huang S., Quan L. (2021). Determination of potential therapeutic targets and prognostic markers of ovarian cancer by bioinformatics analysis. *BioMed Research International*.

[24] Liu J., Lichtenberg T., Hoadley K. A. (2018). An integrated TCGA pan-cancer clinical data resource to drive high-quality survival outcome analytics. *Cell*.

[25] Győrffy B., Surowiak P., Budczies J., Lánczky A. (2013). Online survival analysis software to assess the prognostic value of biomarkers using transcriptomic data in non-small-cell lung cancer. *PLoS One*.

[26] Chen D., Zhang R., Zhang H. (2021). High expression of LUM independently predicts poor prognosis in gastric cancer: a bioinformatics study combining TCGA and GEO datasets. *All Life*.

[27] Huang da W., Sherman B. T., Lempicki R. A. (2009). Systematic and integrative analysis of large gene lists using DAVID bioinformatics resources. *Nature Protocols*.

[28] Hänzelmann S., Castelo R., Guinney J. (2013). GSVA: gene set variation analysis for microarray and RNA-seq data. *BMC Bioinformatics*.

[29] Bindea G., Mlecnik B., Tosolini M. (2013). Spatiotemporal dynamics of intratumoral immune cells reveal the immune landscape in human cancer. *Immunity*.

[30] Bonome T., Levine D. A., Shih J. (2008). A gene signature predicting for survival in suboptimally debulked patients with ovarian cancer. *Cancer research*.

[31] Yoshihara K., Tajima A., Komata D. (2009). Gene expression profiling of advanced-stage serous ovarian cancers distinguishes novel subclasses and implicates ZEB2 in tumor progression and prognosis. *Cancer Science*.

[32] Yu B., Guo X. (2021). Prognostic significance of HOXD4 protein expression in human ovarian cancers. *Iranian Journal of Basic Medical Sciences*.

[33] Sun S., Wang N., Sun Z., Wang X., Cui H. (2019). MiR-5692a promotes proliferation and inhibits apoptosis by targeting HOXD8 in hepatocellular carcinoma. *J BUON*.

[34] Wen D., Wang L., Tan S., Tang R., Xie W., Liu S. (2020). HOXD9 aggravates the development of cervical cancer by transcriptionally activating HMCN1. *Panminerva Medica*.

[35] Mo R. J., Lu J. M., Wan Y. P. (2017). Decreased HoxD10 expression promotes a proliferative and aggressive phenotype in prostate cancer. *Current Molecular Medicine*.

[36] Li S., Zhang J., Zhao Y., Wang F., Chen Y., Fei X. (2018). miR-224 enhances invasion and metastasis by targeting HOXD10 in non-small cell lung cancer cells. *Oncology Letters*.

[37] Wang J., Liu Z., Zhang C. (2021). Abnormal expression of HOXD11 promotes the malignant behavior of glioma cells and leads to poor prognosis of glioma patients. *PeerJ*.

[38] Makiyama K., Hamada J., Takada M. (2005). Aberrant expression of HOX genes in human invasive breast carcinoma. *Oncology Reports*.

[39] Zhong Z. B., Shan M., Qian C. (2015). Prognostic significance of HOXD13 expression in human breast cancer. *International Journal of Clinical and Experimental Pathology*.

[40] Bai Z., Li H., Li C., Sheng C., Zhao X. (2020). Integrated analysis identifies a long non-coding RNAs-messenger RNAs signature for prediction of prognosis in hepatitis B virus-hepatocellular carcinoma patients. *Medicine (Baltimore)*.

[41] Zhang Y., Zhang Q., Cao Z., Huang Y., Cheng S., Pang D. (2018). HOXD3 plays a critical role in breast cancer stemness and drug resistance. *Cellular Physiology and Biochemistry*.

[42] Chen F., Sun G., Peng J. (2016). RNAi-mediated HOXD3 knockdown inhibits growth in human RKO cells. *Oncology Reports*.

[43] Tan Y., Zhang B., Wu T. (2009). Transcriptional inhibiton of Hoxd4 expression by miRNA-10a in human breast cancer cells. *BMC Molecular Biology*.

[44] Mansour M. A., Senga T. (2017). HOXD8 exerts a tumor-suppressing role in colorectal cancer as an apoptotic inducer. *The International Journal of Biochemistry & Cell Biology*.

[45] Zhu H., Dai W., Li J. (2019). HOXD9 promotes the growth, invasion and metastasis of gastric cancer cells by transcriptional activation of RUFY3. *Journal of Experimental & Clinical Cancer Research*.

[46] Xiong R., Yin T., Gao J. L., Yuan Y. F. (2020). HOXD9 activates the TGF-*β*/Smad signaling pathway to promote gastric cancer. *Oncotargets and Therapy*.

[47] Yuan Y. H., Wang H. Y., Lai Y. (2019). Epigenetic inactivation of HOXD10 is associated with human colon cancer via inhibiting the RHOC/AKT/MAPK signaling pathway. *Cell Communication and Signaling*.

[48] Nakayama I., Shibazaki M., Yashima-Abo A. (2013). Loss of HOXD10 expression induced by upregulation of miR-10b accelerates the migration and invasion activities of ovarian cancer cells. *International Journal of Oncology*.

[49] Sharpe D. J., Orr K. S., Moran M. (2014). POU2F1 activity regulates HOXD10 and HOXD11 promoting a proliferative and invasive phenotype in head and neck cancer. *Oncotarget*.

[50] Xu G., Wu Y. L., Li N. (2020). GALNT10 promotes the proliferation and metastatic ability of gastric cancer and reduces 5-fluorouracil sensitivity by activating HOXD13. *European Review for Medical and Pharmacological Sciences*.

[51] Zhang J., Deng M., Tong H. (2020). A novel miR-7156-3p-HOXD13 axis modulates glioma progression by regulating tumor cell stemness. *International Journal of Biological Sciences*.

