# Supplementary material for: Molecular Analysis of Prognosis and Immune Infiltration of Ovarian Cancer Based on Homeobox D Genes
Source: Comput Math Methods Med. 2022 Sep 29;2022:3268386. doi: 10.1155/2022/3268386 (PMC9537619; doi:10.1155/2022/3268386)
Supplement: Supplementary 1 — Figure S1: the prognostic value of mRNA level of HOXD12 in OC patients (Kaplan-Meier plotter). (A) Overall survival of HOXD12 and (B) progression free interval of HOXD12. The HR and log-rank P values are indicated at the right corner of the plot. Log − rank < 0.05 indicates that the difference is statistically significant. [file 3268386.f1.zip › Supplementary figure.docx]

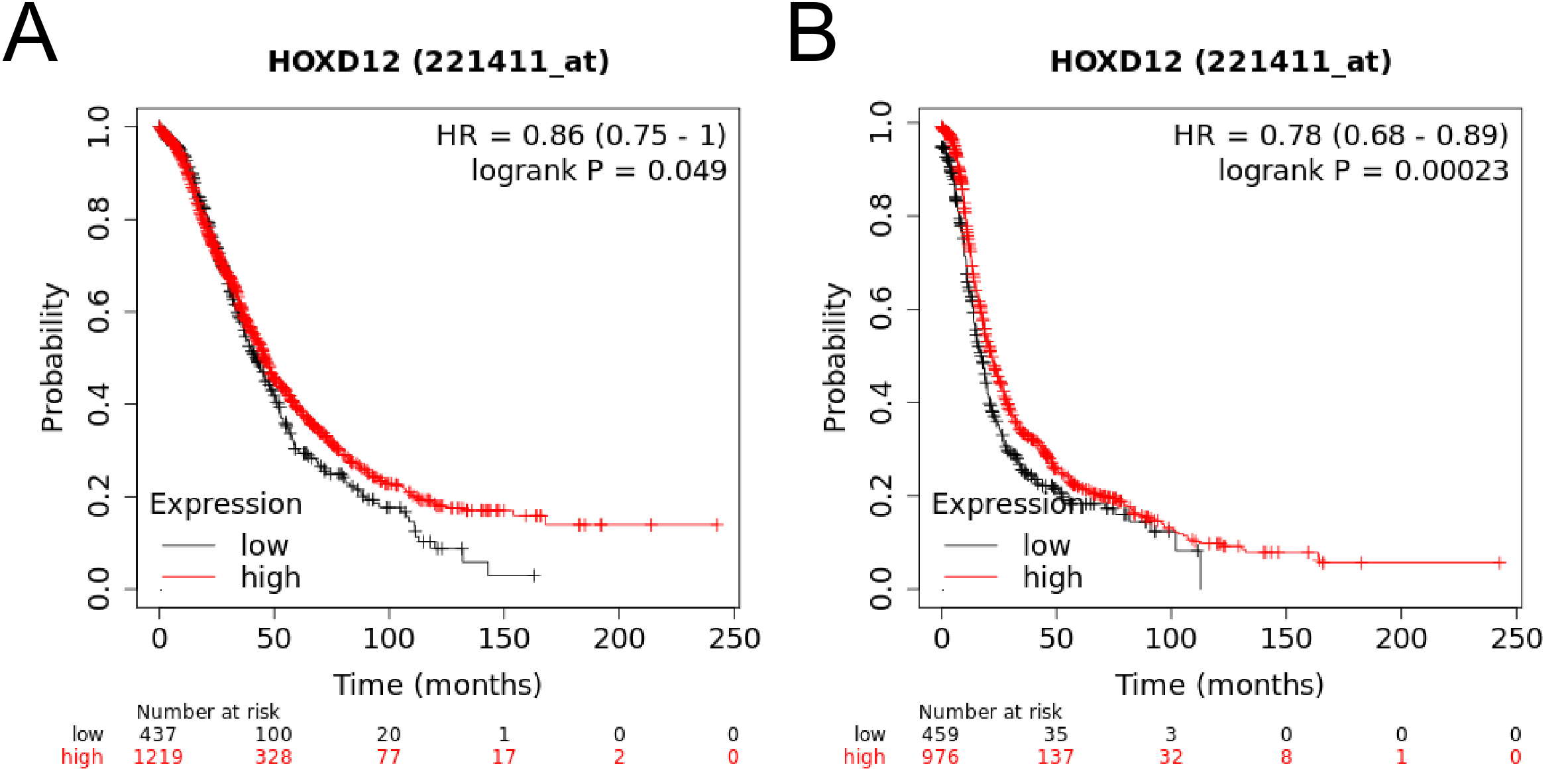


**Figure S1.** The prognostic value of mRNA level of HOXD12 in OC patients (Kaplan-Meier plotter). (A) overall survival of HOXD12, (B) progression free interval of HOXD12. The HR and log-rank P values are indicated at the right corner of the plot. Log-rank < 0.05 indicates that the difference is statistically significant.
