# Supplementary figures and images for: Molecular Analysis of Prognosis and Immune Infiltration of Ovarian Cancer Based on Homeobox D Genes
Source: Comput Math Methods Med. 2022 Sep 29;2022:3268386. doi: 10.1155/2022/3268386 (PMC9537619; doi:10.1155/2022/3268386)

A

HOXD12 (221411\_at)

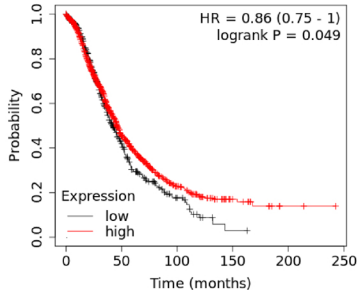

|      | Number at risk |     |    |    |   |
|------|----------------|-----|----|----|---|
| low  | 437            | 100 | 20 | 1  | 0 |
| high | 1219           | 328 | 77 | 17 | 0 |

B

HOXD12 (221411\_at)

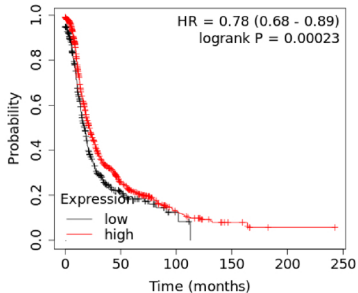

|      | Number at risk |     |    |   |   |
|------|----------------|-----|----|---|---|
| low  | 459            | 35  | 3  | 0 | 0 |
| high | 976            | 137 | 32 | 8 | 1 |

Supplement: Supplementary 1 — Figure S1: the prognostic value of mRNA level of HOXD12 in OC patients (Kaplan-Meier plotter). (A) Overall survival of HOXD12 and (B) progression free interval of HOXD12. The HR and log-rank P values are indicated at the right corner of the plot. Log − rank < 0.05 indicates that the difference is statistically significant. [file 3268386.f1.zip › Figure S1.pdf]
